# Comparison of the coverage and rotation of asymmetrical and symmetrical tibial components: a systematic review and meta-analysis

**DOI:** 10.1186/s12891-024-07466-2

**Published:** 2024-04-26

**Authors:** Ziming Zhang, Tiantian Zhang, Le Zhang, Zehua Chen, Haoming Zhao, Jianjun Kuang, Liang Ou

**Affiliations:** 1 Hunan Academy of Chinese Medicine, 58 Lushan Street, Changsha, 410006 China; 2 Hunan University of Chinese Medicine, Changsha, China; 3 Department of Orthopedics, Orthopedics Hospital of Chinese Medicine Zhuzhou City, Zhuzhou, China

**Keywords:** Total knee arthroplasty, Asymmetric tibial component, Tibial baseplate, Rotational alignment

## Abstract

**Background:**

An optimized fit of the tibial component to the resection platform and correct rotational alignment are critical for successful total knee arthroplasty (TKA). However, there remains controversy regarding the superiority of symmetric tibial component versus asymmetric tibial component. The objective of this systematic review and meta-analysis was to evaluate the current evidence for comparing the coverage and rotation of asymmetrical and symmetrical tibial component.

**Methods:**

We searched potentially relevant studies form PubMed, Web of science, Embase, Cochrane Central Register of Controlled Trials (CENTRAL), and China National Knowledge Infrastructure (CNKI), up to 1 March 2023. Data extraction and quality assessment were performed by two independent reviewers. Meta-analysis was conducted using Review Manager 5.4.

**Results:**

Sixteen articles were identified. Compared to symmetric tibial component, asymmetric tibial component increased the coverage of the proximal tibial cut surface (MD, -2.87; 95%CI, -3.45 to -2.28; *P* < 0.00001), improved the prevalence of tibial baseplate underhang (OR, 0.16; 95%CI, 0.07 to 0.33; *P* < 0.00001) and malrotation (OR, 0.13; 95%CI, 0.02 to 0.90; *P* = 0.04), and reduced the degree of tibial component rotation (MD, -3.11; 95%CI, -5.76 to -0.47; *P* = 0.02). But there was no statistical significance for improving tibial baseplate overhang (OR, 0.58; 95%CI, 0.08 to 3.97; *P* = 0.58). Additionally, no revision had occurred for the two tibial components in the included studies.

**Conclusion:**

The current evidence shows asymmetric tibial component offer advantages in terms of coverage and rotation compared with symmetric tibial component in TKA.

**Supplementary Information:**

The online version contains supplementary material available at 10.1186/s12891-024-07466-2.

## Background

Total knee arthroplasty (TKA), as a commonly performed elective orthopaedic surgery, provides patients with considerable medium- and long-term benefits in terms of quality of life, pain relief and function [1]. However, dissatisfaction after knee arthroplasty remains around 15 to 20% [2]. Numerous factors have an influence on clinical outcomes of TKA, among which the choice and ideal positioning of tibial prosthesis are particularly critical. Currently, there are two designs for tibial component available: symmetric tibial component (STC) and asymmetric tibial component (ATC).

Precise matching of the tibial component and resected plateau and proper rotational alignment of the tibial component are essential for successful TKA. Several studies have concluded that STC may not be suitable for all races [3]. Recently, asymmetric and even markedly anatomical designs have been introduced to improve the bony coverage and rotational alignment in TKA, out of consideration for the asymmetric proximal tibial cut surface.

How much does the ATC improve the bony coverage remains elusive. The proponents of ATC often argue that the use of ATC has many advantages, including better tibial coverage with less overhang, easier to place with decreased internal rotation of the tibial component, and longer implant longevity [4, 5]. On the contrary, some evidence supports that there is small improvement of tibial coverage compared with the STC, and even the STC is more effective in providing the ideal tibial rotation [6]. Objectively, between the two tibial base designs in radiographic and clinical outcomes, the superiority of one to the other is still controversial. Therefore, we undertook this systematic review and meta-analysis to compare the coverage and rotation, as well as clinical outcomes, of the STC and ATC.

## Methods

### Literature search

This systematic review and meta-analysis was performed in accordance with the Preferred Reporting Items for Systematic Reviews and Meta-analyses (PRISMA) guidelines [7] (Additional files 1 and 2). We had registered this review in the International Prospective Register of Systematic Reviews (PROSPERO, identifier CRD42023418486). We searched potentially relevant studies form PubMed, Web of science, Embase, Cochrane Central Register of Controlled Trials (CENTRAL), and China National Knowledge Infrastructure (CNKI), up to 1 March 2023. The following search terms: total knee arthroplasty, TKA, asymmetric, anatomic, tibial baseplate, tibial tray, and tibial component, etc., were used to retrieved by means of a combination of Mesh terms and free terms. In addition, we performed a manual search for references of included studies. Detailed search strategies are showed in Additional files 3.

### Eligibility criteria

A comparative study, including randomized controlled trial (RCT) or cohort study, of ATC versus STC superimposed in the tibial section was considered for inclusion. The included studies should meet the following criteria: (1) patients who was performed with TKA surgery or just with virtual surgery for imaging studies; (2) comparators for ATC versus STC; (3) outcomes including coverage and/or rotation of tibial prosthesis, or revision rate, or clinical outcomes. Furthermore, studies would be excluded if met any of the following criteria: revision TKA, asymmetrical polyethylene, finite element analysis, animal or cadaveric studies, protocols, case reports, reviews, and full-text or data unavailable articles.

### Data extraction

First, two independent reviewers, according to the above search strategy and inclusion criteria, followed the standard process for literature screening which was consisted of removing duplicate studies, eliminating obviously irrelevant studies by reading the titles and abstracts, and including eligibility studies after reading the full text. Subsequently, the two reviewers extracted the following information from included studies: primary author, publication year, country of study, study design, number of patients and knees, age and gender, type of prosthesis, length of follow-up, and outcomes. Ultimately, any disagreement in the above process would be resolved by consultation with the third reviewer.

### Outcomes of interest

We mainly focused on the coverage and rotation of tibial prosthesis which included the coverage rate, underhang, and overhang, and malrotation and rotation degree of tibial prosthesis, respectively. Besides, we compared the revision rate and clinical outcome measures of the two tibial components. Coverage rate was defined as the total cross-sectional area of the appropriately sized tray minus any tray overhang, divided by the total cross-sectional area of the tibial surface. Overhang was defined as the absence of tibia bone below the base plate on immediate, and underhang was defined as exposure of the tibial cut surface. Generally, overhang of less than 1 mm and underhang of less than 2 mm was regarded as an optimal fit. Therefore, an overhang of over 2 mm was regarded as absolute overhang and an underhang of over 3 mm was regarded as absolute underhang, which were both unacceptable. Malrotation was defined as the implant axis being over 5°of deviation from the axis of neutral tibial rotational.

### Methodology assessment

Independently, two investigators assessed the methodological quality of RCTs adhering to the standards advised by the Cochrane Collaboration risk of bias table [8]. The risk of bias was evaluated from the following seven aspects: random sequence generation, allocation concealment, blinding of participants and personnel, blinding of outcome assessment, integrity of results data, selective reporting of results, and other bias. Moreover, Newcastle–Ottawa scale was utilized for evaluating the methodological quality of cohort studies, which includes three aspects: population selection, comparability, and outcome [9].

### Statistical analysis

Meta-analyses were conducted using RevMan (version 5.4, Cochrane Collaboration). In this review, continuous variables such as percentage of coverage and rotation degree of tibial prosthesis were pooled and analyzed mean difference (MD) with 95% confidence intervals (95% CIs), and dichotomous variables including underhang, overhang, and malrotation were pooled by odds ratio (OR) with 95% CIs. Heterogeneity was evaluated by Higgins *I*^2^ statistic which ranges from 0 to 100%. An *I*^2^ > 50% indicates substantial heterogeneity. The random-effect model was applied as we have identified clinical and methodological heterogeneity among studies. Subgroup analyses were performed to identify potential determinants of efficacy. Sensitivity analysis was also conducted to explore potential sources of heterogeneity between studies. Additionally, funnel plots were constructed, if possible, to evaluate publication bias. A *P* value threshold of 0.05 was used to determine statistical significance.

## Results

### Search results

**Fig. 1:**
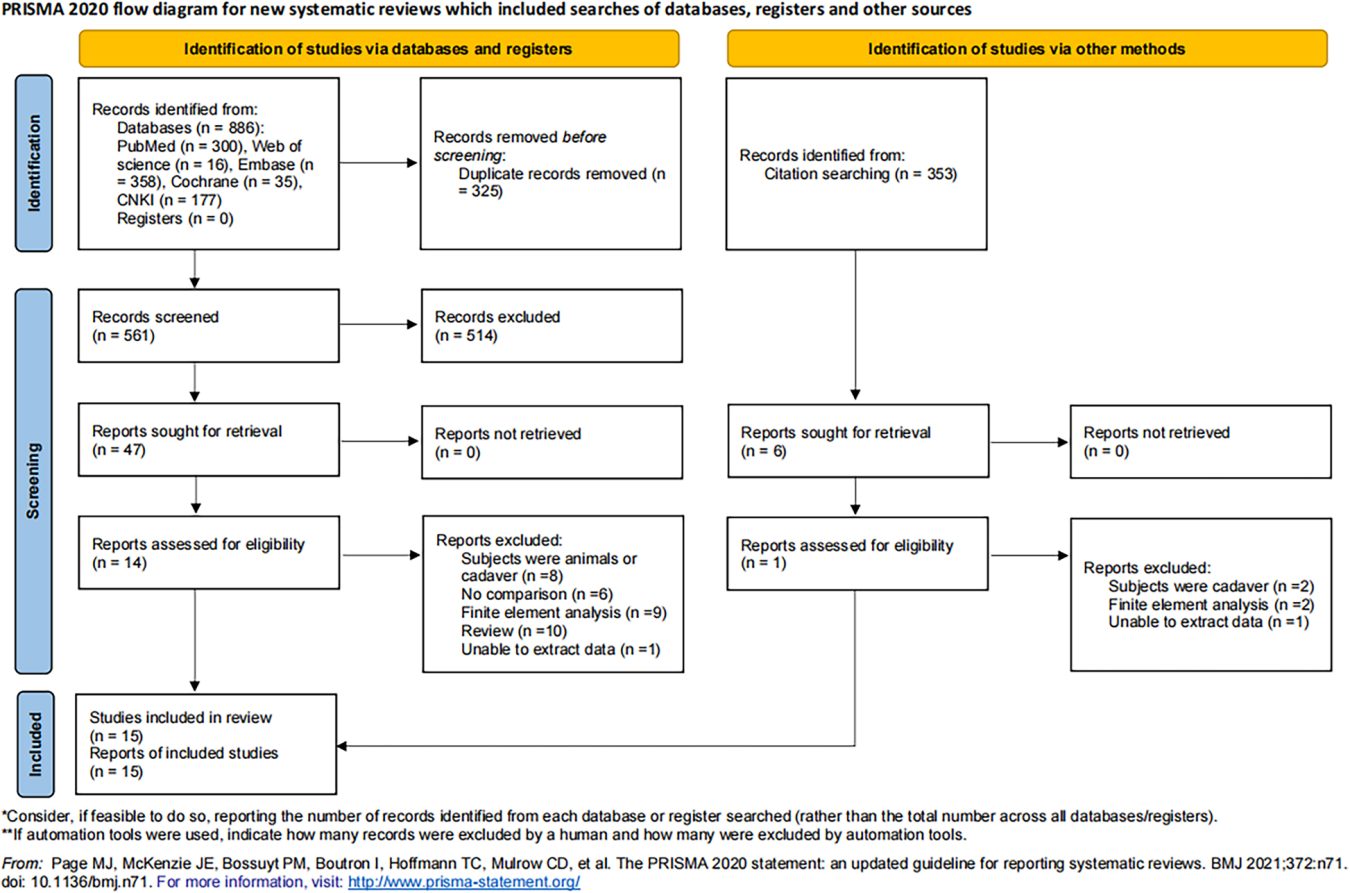
PRISMA flow diagram of literature search

A total of 1175 potentially eligible records were identified via databases. After removing 525 duplicate records, 650 publications underwent title and abstract screening and 603 were excluded. Full texts of 47 records were reviewed, and 14 studies assessed for eligibility. Furthermore, we identified 353 references from the all including studies and 2 reference assessed for eligibility. Eventually, the present review included 16 articles for analysis [10–25] (Table 1). The detailed selection flow is shown in Fig. 1.

**Table 1:** Summary of studies included in meta-analysis

Study	Country	Design	Prosthesis types		Operation type	Patients/Knees	Male/Female	Age(years)	Outcomes	Mean Follow-up(months)
			**Asymmetrical**	**Symmetric**						
Koster LA et al., 2021 [10]	Netherlands	RCT	Persona	NexGen	Actual TKA	31/31 38/38	13/19 16/22	63.8 ± 12.0 67.3 ± 8.0	Revision rate	24
Minoda Y et al., 2017 [11]	Japan	RCT	Advance medial pivot	Evolution medial pivot	Actual TKA	31/31^a^	2/29^a^	69 ± 13^a^	Overhang	None
Indelli PF et al., 2015 [12]	USA	RCT	Persona	NexGen	Actual TKA	40/40 40/40	16/24 15/25	71(66–80)^b^ 72(60–81)^b^	Rotation of the tibial component; Revision rate; Clinical outcomes (Anterior knee pain, Oxford, ROM, Overall satisfaction rate, Survivorship)	24
Cho BW et al., 2020 [13]	Korea	Retrospective cohort	Persona	NexGen	Actual TKA	59/78 58/74	9/50 4/54	71.2 ± 5.7 71.1 ± 6.2	Underhang; Overhang; Revision rate; Clinical outcomes (VAS, KSS, WOMAC)	31.9 ± 5.8 33.9 ± 11.5
Rhee SJ et al., 2018 [14]	Korea	RCT	Persona	NexGen	Actual TKA	NR/50 NR/51	4/46 7/44	67.3 ± 7.3 68.7 ± 7.2	Rotation of the tibial component	0.5
Jin C et al., 2016 [15]	Korea	Retrospective cohort	Persona	NexGen	Actual TKA	100/100^a^	9/91^a^	71(56–83)^a,b^	Coverage rate; Underhang; Overhang;	None
Shaet s al, 2015 [16]	India	RCT	Genesis II	NexGen	Virtual TKA	150/300^a^	63/87^a^	35.5(18–50)^a,b^	Coverage rate; Underhang; Overhang;	None
Meier M et al., 2018 [17]	USA	Retrospective cohort	Persona	Sigma	Virtual TKA	100/100^a^	50/50^a^	65.6(49–93) ^a,b^	Coverage rate	None
Stulberg SD et al., 2015 [18]	USA	Retrospective cohort	Persona	NexGen	Virtual TKA	91/100^a^	29/62^a^	66.4(41–84) ^a,b^	Coverage rate; Rotation of the tibial component; Malrotation	None
Ma Y et al., 2017 [19]	Japan	Retrospective cohort	Persona	NexGen	Virtual TKA	77/77^a^	15/62^a^	NR	Overhang; Rotation of the tibial component	None
Martin S et al., 2014 [20]	USA	Retrospective cohort	NR	NR	Virtual TKA	30/30^a^	NR	NR	Coverage rate; Rotation of the tibial component; Malrotation	None
Wernecke GC et al., 2012 [21]	Australia	Retrospective cohort	Genesis II	NexGen	Virtual TKA	101/101^a^	74/27^a^	32(17–60) ^a,b^	Coverage rate; Underhang; Overhang;	None
Clary C et al., 2014 [22]	USA	Retrospective cohort	Genesis II	Sigma	Virtual TKA	14,791/14791^a^	NR	NR	Coverage rate	None
Miyatake N et al., 2016 [23]	Japan	Retrospective cohort	Genesis II	NexGen	Actual TKA	NR/92 NR/586	9/83 111/475	73.7 ± 6.7^a^	Underhang	None
Maciag BM et al., 2021 [24]	Poland	Retrospective cohort	Persona	NexGen	Actual TKA	39/39 34/34	13/26 12/22	68.6 ± 6.3 69.5 ± 5.7	Underhang; Overhang;	1.5
Bizzozero P et al., 2018 [25]	France	Retrospective cohort	Persona	NexGen	Actual TKA	33/33 33/33	15/17 15/17	77(57–92)^b^ 75(66–89)^b^	Coverage rate; Overhang; Rotation of the tibial component; Malrotation; Clinical outcomes (KSS)	44 ± 5.4 46 ± 5.7

*RCT* Randomized controlled trial, *NR* No record, *KSS* Knee scociety score, *ROM* Range of motion, *VAS* Visual analogue scale, *WOMAC* Western Ontario and McMaster Universities Osteoarthritis Index, *TKA* Total knee arthroplasty

^a^Values are presented as the same patients in the crossover design

^b^Values are presented as mean(range)

### Study characteristics

In total, 5 RCTs and 11 cohort studies were included. Five studies were from USA [12, 17, 18, 20, 22], three studies each from Korea [13–15] and Japan [11, 19, 23], and each one study from France [25], Netherlands [10], Australia [21], Poland [24] and India [16]. Among 16 studies, 9 studies actually finished the surgery [10–15, 23–25], and the remaining 7 studies just simulated the placement of the prosthesis using imaging software [16–22]. On average, patients were a higher proportion of female than male. For selection of asymmetric prostheses, ten studies used Persona [10, 12–15, 17–19, 24, 25], four studies chose Genesis II [16, 21–23], one study picked the Evolution medial pivot produced by MicroPort Orthopedics [11] and one study did not tell direct us which prosthesis used [20]. NexGen was one of the most used symmetrical prostheses. In addition, four studies provided the hip-knee-ankle angle and showed the average varus angle of the affected knee ranges from 3.2–9.9° [13, 14, 19, 25].

### Quality assessment

**Fig. 2:**
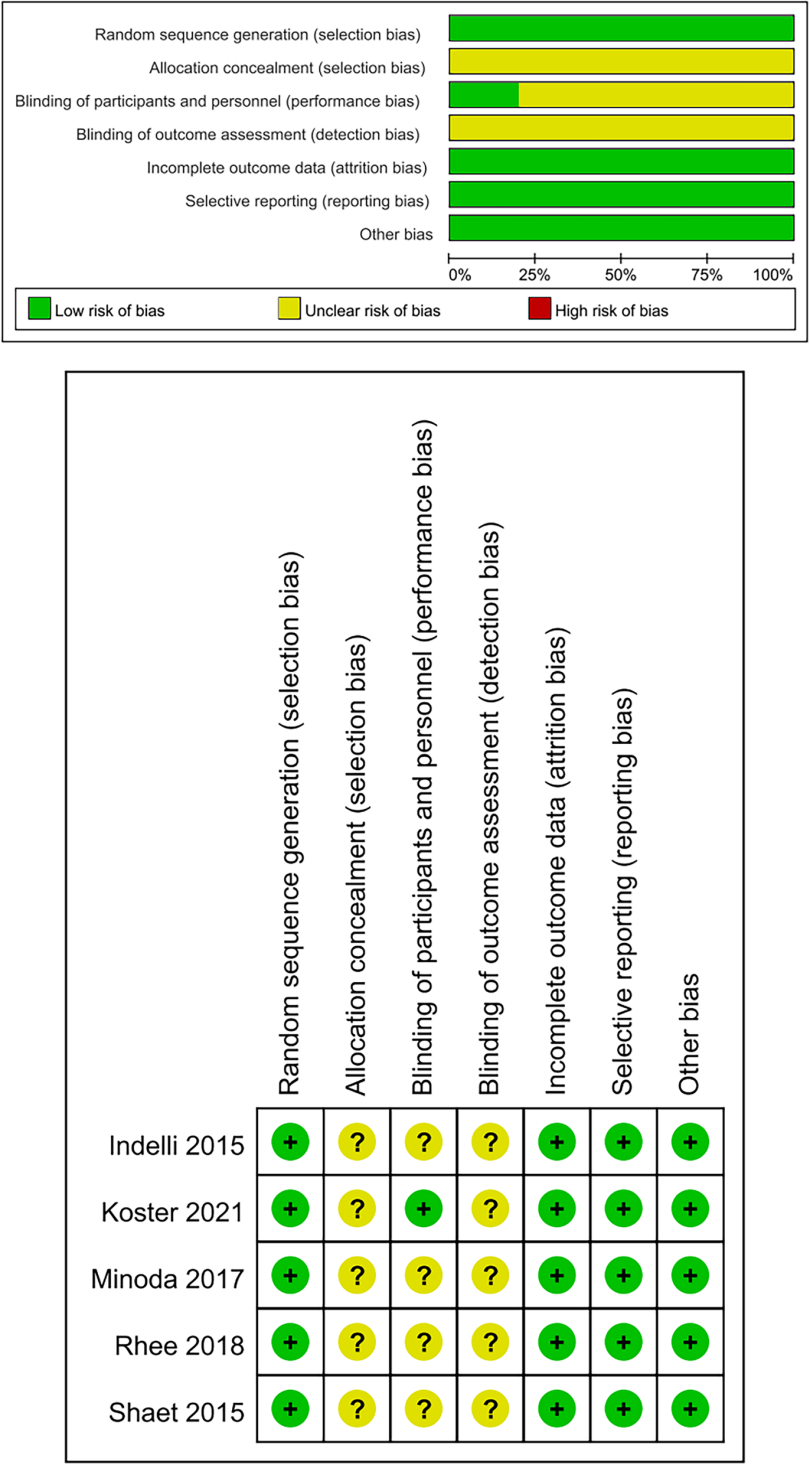
Risk of bias graph

RCTs and cohort studies were assessed by Cochrane Collaboration risk of bias table and Newcastle–Ottawa scale, respectively. Of the five RCTs [10–12, 14, 16], all studies showed a low risk for random sequence generation, incomplete outcome data, selective reporting, and other bias and presented an unclear risk for allocation concealment and blinding of outcome assessment. One study described as patient-blinded [10], exhibited a low risk for allocation concealment and others were recognized as an unclear risk. Of the eleven cohort studies [13, 15, 17–25], eight scored 9 points and three scored 7 points. Hence, the studies were of a relatively high quality. Detailed results are displayed in Table 2 and Fig. 2.

**Table 2:** Quality assessment of included studies according to the Newcastle–Ottawa scale

Study	Selection				Comparability		Exposure			Total score
	Representativeness of the exposed cohort	Selection of the non-exposed cohort	Ascertainment of exposures	Demonstration that outcome of interest was not present at start of study	Comparability of cohorts on the basis of the design or analysis	Study controls for any additional factor	Assessment of outcome	Was follow-up long enough for outcomes to occur	Adequacy of follow-up of cohorts	
Cho BW et al., 2020 [13]	1	1	1	1	1	1	1	1	0	8
Jin C et al., 2016 [15]	1	1	1	1	1	1	1	1	1	9
Meier M et al., 2018 [17]	1	1	1	1	1	1	1	1	1	9
Stulberg SD et al., 2015 [18]	1	1	1	1	1	1	1	1	1	9
Ma Y et al., 2017 [19]	1	1	1	1	1	1	1	1	1	9
Martin S et al., 2014 [20]	1	1	1	1	1	1	1	1	1	9
Wernecke GC et al., 2012 [21]	1	1	1	1	1	1	1	1	1	9
Clary C et al., 2014 [22]	1	1	1	1	1	1	1	1	1	9
Miyatake N et al., 2016 [23]	1	1	1	1	1	1	1	0	1	8
Maciag BM et al., 2021 [24]	1	1	1	1	1	1	1	1	0	8
Bizzozero P et al., 2018 [25]	1	1	1	1	1	1	1	1	1	9

### Coverage

#### Coverage rate

**Fig. 3:**
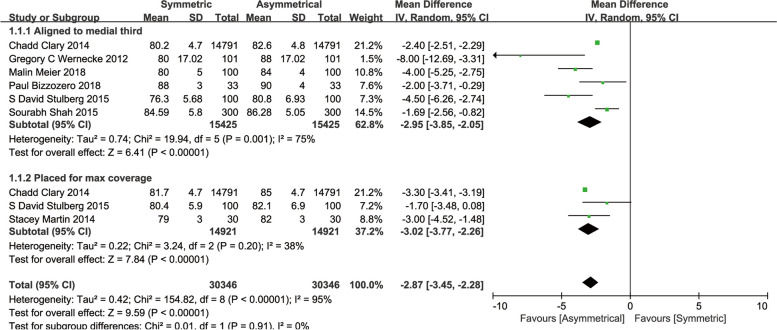
Meta-analysis and forest plot for coverage rate

Seven studies compared the tibial bone coverage of two tray designs [16–18, 20–22, 25]. An overall meta-analysis showed the ATC achieved significantly more tibial coverage than the STC did (MD, -2.87; 95%CI, -3.45 to -2.28; *P* < 0.00001). A further subgroup analysis indicated the rate of tibial coverage of ATC was significantly higher than STC, whether tibial prosthesis aligned to the medial third of the tubercle (MD, -2.95; 95%CI, -3.85 to -2.05; *P* < 0.00001) or placed to maximum coverage (MD, -3.02; 95%CI, -3.77 to -2.26; *P* < 0.00001) (Fig. 3). Furthermore, a subgroup analysis of operation type, being divided into actual TKA or simulated TKA, showed that ATC presented a better coverage rate both in actual TKA (MD, -2.00; 95%CI, -3.71 to -0.29; *P* = 0.02) and in simulated TKA (MD, -2.94; 95%CI, -3.55 to -2.33; *P* < 0.00001) (Supplementary figure S1).

#### Underhang

**Fig. 4:**
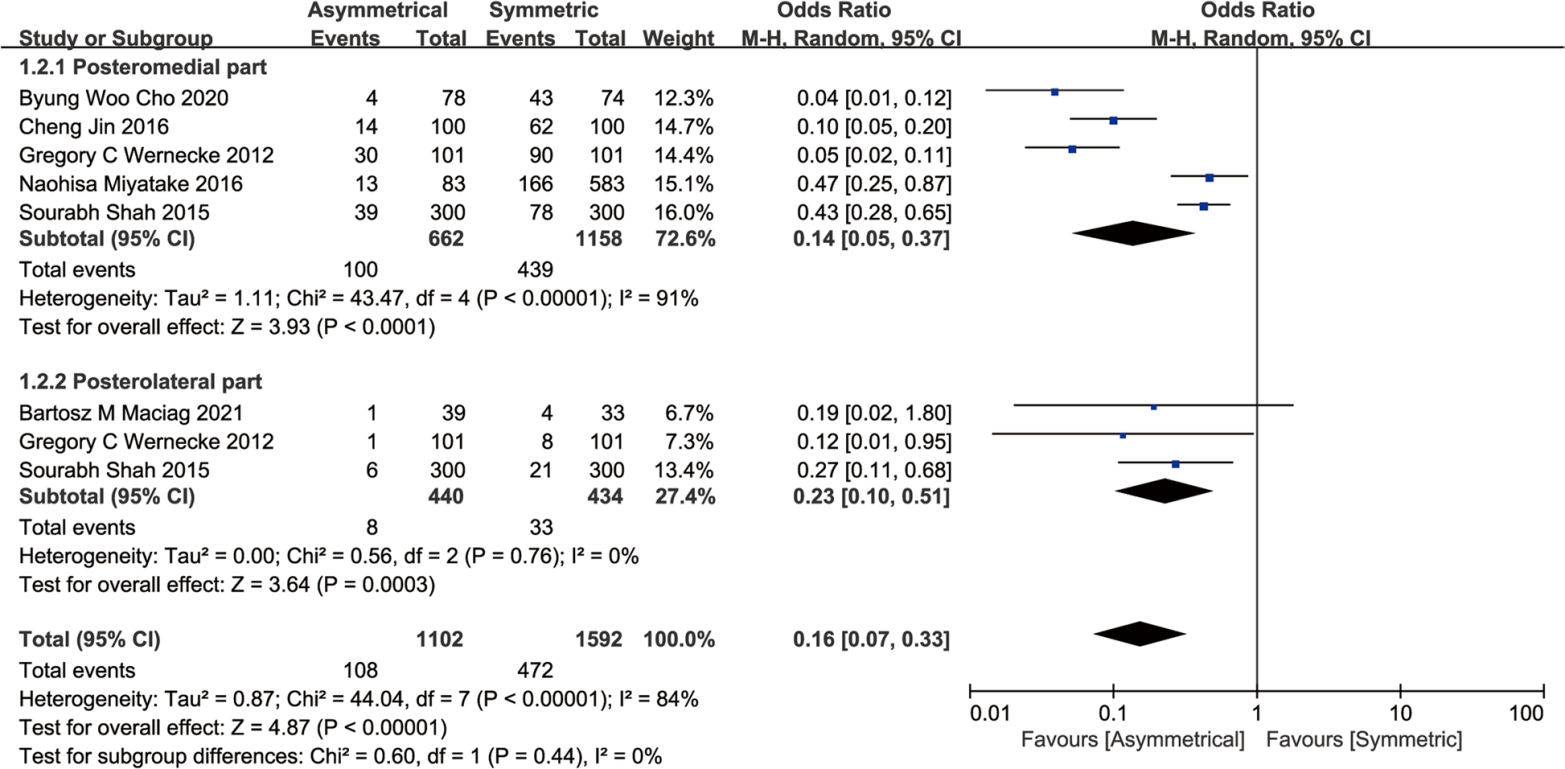
Meta-analysis and forest plot for underhang

An overall meta-analysis of six studies suggested the ATC had significant improvement for the tibial baseplate underhang compared the STC (OR, 0.16; 95%CI, 0.07 to 0.33; *P* < 0.00001) [13, 15, 16, 21, 23, 24]. A further subgroup analysis found the prevalence of posteromedial tibial baseplate underhang, as well as posterolateral, was lower with the ATC compared to the ATC, (OR, 0.14; 95%CI, 0.05 to 0.37; *P* < 0.0001) and (OR, 0.23; 95%CI, 0.10 to 0.51; *P* = 0.0003), respectively (Fig. 4).

#### Overhang

**Fig. 5:**
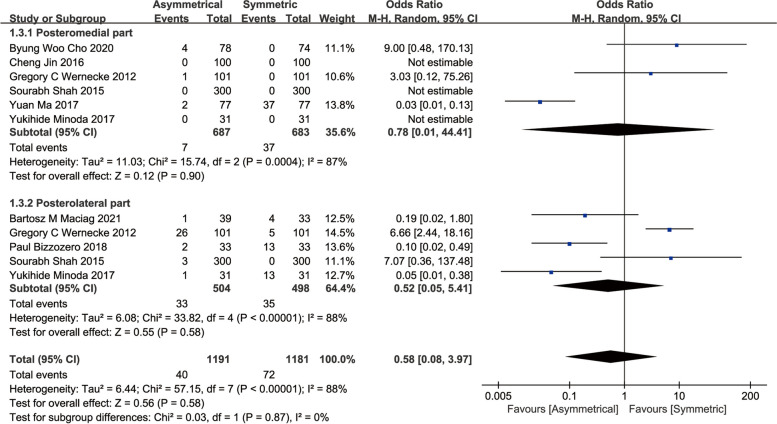
Meta-analysis and forest plot for overhang

Eight studies measured the tibial baseplate overhang, and no significant differences was found between the two tibial designs (OR, 0.58; 95%CI, 0.08 to 3.97; *P* = 0.58) [11, 13, 15, 16, 19, 21, 24, 25], as well as with subgroup analysis results for posteromedial and posterolateral tibial baseplate overhang, (OR, 0.78; 95%CI, 0.01 to 44.41; *P* = 0.90) and (OR, 0.52; 95%CI, 0.05 to 5.42; *P* = 0.58), respectively (Fig. 5).

### Rotation

#### Malrotation

**Fig. 6:**
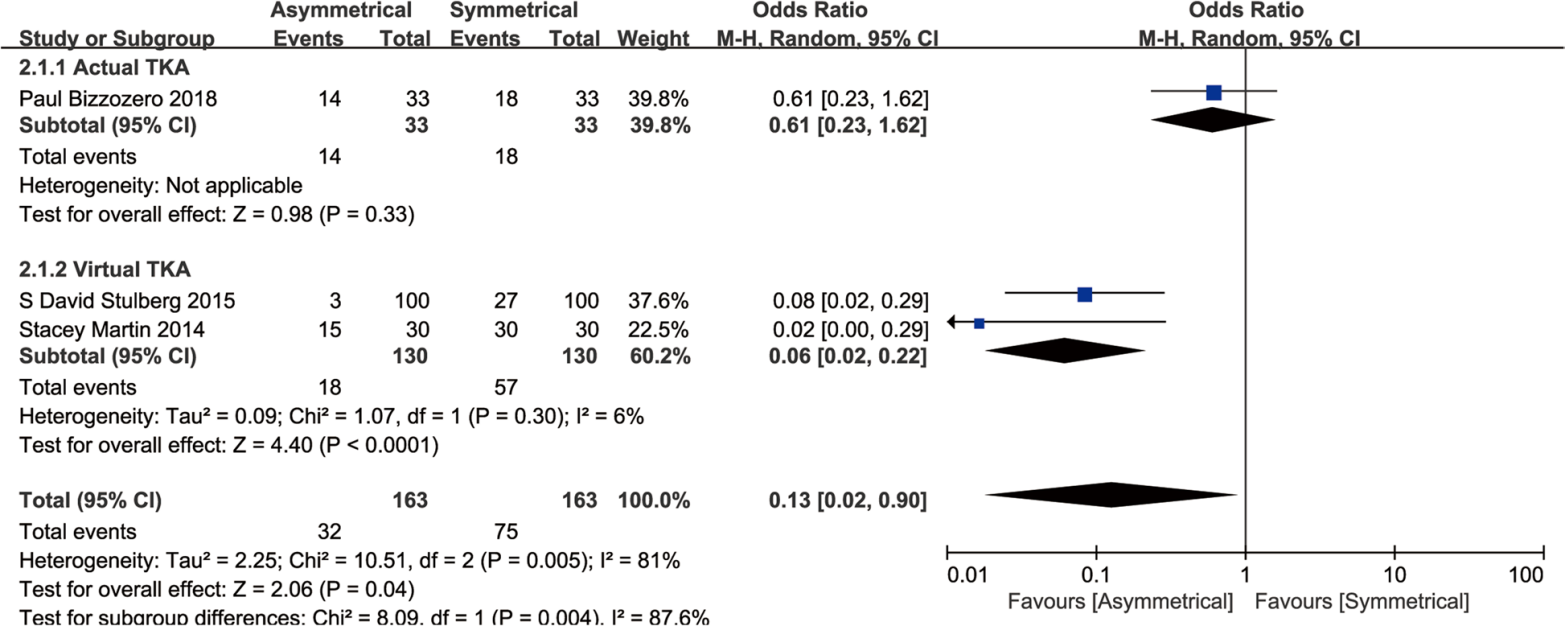
Meta-analysis and forest plot for malrotation

Only three studies reported the tibial component malrotation, and a meta-analysis revealed that there was a lower malrotation with the ATC compared to the STC (OR, 0.13; 95%CI, 0.02 to 0.90; *P* = 0.04) [18, 20, 25]. However, a subgroup analysis indicated that ATC presented a better rotational alignment in simulated TKA (OR, 0.61; 95%CI, 0.23 to 1.62; *P* = 0.33), but did not reveal any significant differences in actual TKA (OR, 0.06; 95%CI, 0.02 to 0.22; *P* < 0.0001) (Fig. 6).

#### Degree of rotation

**Fig. 7:**
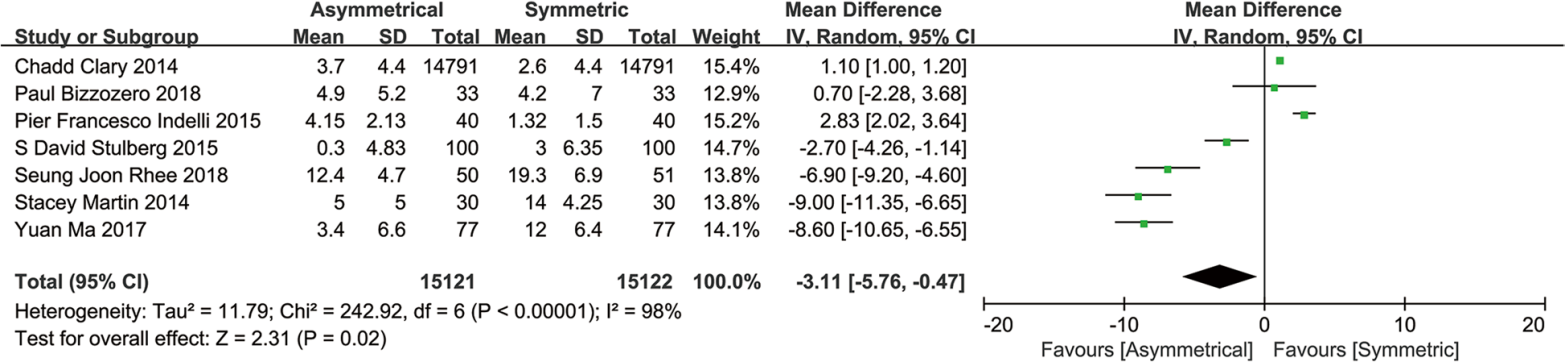
Meta-analysis and forest plot for degree of rotation

A meta-analysis of seven studies, six placing for maximum coverage and one positioning along the Install line, showed the ATC generated a smaller degree of rotation than the STC (MD, -3.11; 95%CI, -5.76 to -0.47; *P* = 0.02) [12, 14, 18–20, 22, 25] (Fig. 7).

#### Revision rate

Only four studies had followed for more than 2 years [10, 12, 13, 15], and three out of them indicated that no revision had occurred in each group.

#### Clinical outcomes

Only two studies examined KSS Score, and no significant difference was found between the two tibial components [13, 25]. Only one study reported ATC had a statistically significant reduction in postoperative anterior knee and had a larger average ROM [12]. And no significant difference in VAS, WOMAC, Oxford Score, overall satisfaction rate, and survivorship in two years was found between the two designs in two different studies [12, 13].

#### Publication bias

Because of the number of studies including for meta-analysis not exceeding 10, no funnel plot analysis is necessary.

## Discussion

A suitable tibial component is particularly important for TKA. Despite STCs are used for the vast majority of patients undergoing TKA, current TKA designs do not always provide correct kinematics for the native joint and thus further optimizations to implant designs seem desirable. In general, the indication for using asymptomatic and symmetrical components are the same for the vast majority of patients receiving TKA, but not identical. Therefore, when choosing between the different types of tibial prostheses, the surgeon will personalize the choice based on the patient's specific situation and needs in order to obtain the best possible surgical results and post-operative outcome. In this meta-analysis, a newly introduced ATC exhibits the advantages of anatomical design. The findings in this study suggested that ATC increased the coverage of the proximal tibial cut surface and reduced the prevalence of tibial baseplate underhang. But there was no statistical significance for improving tibial baseplate overhang. Meanwhile, we found that ATC had a smaller degree of rotation, which would attribute to a lower rate of component malrotation. Additionally, no revision had occurred in each tibial component. Of note, we did not conduct a meta-analysis for clinical outcomes, because the number of studies reporting these outcomes was less than two.

An optimized fit at the tibial plateau and correct rotational alignment may result in better outcomes after TKA [26]. For tibial component coverage, matching the resected bony surfaces as much as possible, neither underhang nor overhang, is regarded as the optimal fit. Both underhang as well as excess overhang have been found to lead to adverse outcomes, such as component subsidence, long-term aseptic loosening, soft-tissue irritation, and pain [27]. Morphologically, the human tibial component is inherently asymmetrical, with the medial plateau slightly larger than the lateral [28]. Accordingly, the use of STC often leads to medial tibial plateau anterior and posterior underhang and posterolateral overhang [29]. An earlier systematic review, investigated the clinical outcomes of ATC, among which most of the included studies were retrospective cohort studies and case series, and only 2 RCTs comparing ATC and STC. Due to the low quality of the included studies, the review drew a conclusion with low level evidence that ATC improved tibial coverage and underhang [30], which was consistent with the results of our study. The lower underhang allows the prosthesis to better fit the outer edge of the tibia, and consequently reduce bone loss and osteophyte formation. Nevertheless, the literature data about tibial baseplate overhang are nonetheless controversial. Bonnin found a lateral overhang in 87% of patients operated in his series with a symmetrical tibial tray [31]. Some studies suggested that ATC have been identified to optimize coverage and avoid overhang [4, 32]. In this review, we performed subgroup analysis for underhang and overhang between posteromedial and posterolateral tibial plateau, and found that the prevalence of posteromedial underhang, as well as posterolateral, was lower with the ATC compared to the STC, and no significant differences for posteromedial and posterolateral plateau overhang. Generally speaking, female tibias were smaller in size as compared to males. Of the 16 included studies, only Sourabh Shah DNB. et al. in their study observed the gender differences with respect to the coverage of the two prostheses, and they found that total tibial surface coverage was more for females as compared to males, for both ATC and STC [16].

Another significant cause of TKA failure is tibial component malrotation, which results in pain, stiffness and early revision after TKA [33]. Rotation of the tibial component seemed essential to us in order to optimize the prosthetic kinematics and the patella tracking. However, it is still controversial with regard to the tibial rotational alignment. Based on previous researches, the Insall line has excessive external rotation tendency. Although Akagi line is the most recognized anatomical axis at present, it still has a certain tendency of internal rotation. Additionally, one of the included studies measured the rotational alignment of tibial baseplate with respect to the surgical transepicondylar axis [12]. A retrospective study believed external rotation might be helpful, and recommended that the tibial component be placed with the rotational alignment of 2–5° external rotation [34]. One recent study also found moderate external rotation could improve the kinematics after TKA [35]. In this review, an angle of rotation outside from -5° and 5° was defined as malrotation in the three included studies. And we found the ATC to maximize coverage while preserving rotation within 5° in a greater proportion of cases compared to the STC. In current study, we pooled seven studies for comparing the degree of rotation between two designs. Except for a study by Bizzozero P et al. which paid particular attention to positioning the implant along the Insall line [25], six out of seven studies rotated to max coverage. It indicates that even if the tibial component placed with reference to the standard rotation alignment, it still appears malrotation. Meanwhile, it also shows the ATC optimized the relationship between coverage and rotation.

Our study was not without limitations. First, there were few RCTs included, so we included relevant cohort studies. Therefore, some conclusions should be considered preliminary. Second, because the present study focused primarily on the coverage and rotational alignment of the two tibial components, we included some studies preforming with virtual TKA or anthropometric study using CT and MRI technology, even though the patients in these studies did not underwent TKA. This may result in methodological heterogeneity to some extent, so we did not conduct further sensitivity analysis to explore the source of heterogeneity. Thirdly, we cannot conduct a subgroup analysis to observe whether the differences vary by ethnicity, because the some of the patients in the studies came from different continents, and the differences between different races did not be analyzed in the included studies. Finally, limited by the number of studies reporting postoperative clinical outcomes, our meta-analysis did not find that the ATC was superior to the STC in terms of clinical results. Good radiological results in turn may be responsible for clinical outcomes to some extent, but it would not completely translate into a significant improvement in longevity of prosthesis or functional outcomes. Because there are many factors that affect the clinical outcome, such as postoperative rehabilitation measures, patient's tolerance to pain and patient's physical condition.

## Conclusion

The results of our study are in favor of the use of the ATC allowing to significantly improve coverage and rotation, and reduce the number of underhang, without increasing overhang. However, the evidence for clinical outcomes supporting the ATC is insufficient. Therefore, further research is needed to compared the postoperative functional outcomes for these two different tibial tray designs.

### Supplementary Information

**Supplementary Material 1.**

**Supplementary Material 2.**

**Supplementary Material 3.**

**Supplementary Material 4.**

## Data Availability

The datasets used during the current study are available from the corresponding author on reasonable request.

## Abbreviations

TKA: Total knee arthroplasty
STC: Symmetric tibial component
ATC: Asymmetric tibial component
ROM: Range of motion
VAS: Visual analogue scale
WOMAC: Western Ontario and McMaster Universities Osteoarthritis Index
