# Supplementary material for: Comparison of the coverage and rotation of asymmetrical and symmetrical tibial components: a systematic review and meta-analysis
Source: BMC Musculoskelet Disord. 2024 Apr 26;25:336. doi: 10.1186/s12891-024-07466-2 (PMC11046884; doi:10.1186/s12891-024-07466-2)
Supplement: Supplementary file 3 — Supplementary Material 3. [file 12891_2024_7466_MOESM3_ESM.docx]

# The search strategy in this review.

**（1）The search strategy used for PubMed:**

| Search number | Query | Results |
| --- | --- | --- |
| 6 | (((asymmetric[Title/Abstract]) OR (anatomic*[Title/Abstract])) AND (((((tibial baseplate[Title/Abstract]) OR (tibial base plate[Title/Abstract])) OR (tibial tray[Title/Abstract])) OR (tibial component*[Title/Abstract])) OR (tibial prosthesis[Title/Abstract]))) AND (("Arthroplasty, Replacement, Knee"[Mesh]) OR ((((total knee arthroplasty[Title/Abstract]) OR (Knee Arthroplasty[Title/Abstract])) OR (Knee Replacement[Title/Abstract])) OR (TKA[Title/Abstract]))) | 300 |
| 5 | ("Arthroplasty, Replacement, Knee"[Mesh]) OR ((((total knee arthroplasty[Title/Abstract]) OR (Knee Arthroplasty[Title/Abstract])) OR (Knee Replacement[Title/Abstract])) OR (TKA[Title/Abstract])) | 43,922 |
| 4 | ((((tibial baseplate[Title/Abstract]) OR (tibial base plate[Title/Abstract])) OR (tibial tray[Title/Abstract])) OR (tibial component*[Title/Abstract])) OR (tibial prosthesis[Title/Abstract]) | 3,244 |
| 3 | (asymmetric[Title/Abstract]) OR (anatomic*[Title/Abstract]) | 389,417 |
| 2 | (((total knee arthroplasty[Title/Abstract]) OR (Knee Arthroplasty[Title/Abstract])) OR (Knee Replacement[Title/Abstract])) OR (TKA[Title/Abstract]) | 39,039 |
| 1 | "Arthroplasty, Replacement, Knee"[Mesh] | 30,598 |

**（2）The search strategy used for Embase:**

#6 #3 AND #4 AND #5 358

#5 #1 OR #2 53664

#4 'tibial baseplate':ab,kw,ti OR 'tibial base plate':ab,kw,ti OR 'tibial tray':ab,kw,ti OR 'tibial component':ab,kw,ti OR 'tibial prosthesis':ab,kw,ti 3576

#3 asymmetric:ab,kw,ti OR anatomic*:ab,kw,ti 503089

#2 'total knee arthroplasty':ab,kw,ti OR 'knee arthroplasty':ab,kw,ti OR 'knee replacement':ab,kw,ti OR tka:ab,kw,ti 47671

#1 'total knee arthroplasty'/exp OR 'total knee arthroplasty' 41724

**（3）The search strategy used for Web of Science:**

#4 #1 AND #2 AND #3 304

#3 TS = (tibial tray OR tibial component* OR tibial prosthesis) 7,616

#2 TS = (asymmetric OR anatomical) 532,245

#1 TS = (total knee arthroplasty OR Knee Arthroplasty OR Knee Replacement OR TKA) 56,529

**（4）The search strategy used for CENTRAL:**

#6 #3 AND #4 AND #5 35

#5 (tibial tray):ti,ab,kw OR (tibial component*):ti,ab,kw OR (tibial prosthesis):ti,ab,kw 798

#4 (asymmetric):ti,ab,kw OR (anatomical):ti,ab,kw 13432

#3 #1 OR #2 10595

#2 (total knee arthroplasty):ti,ab,kw OR (Knee Arthroplasty):ti,ab,kw OR (Knee Replacement):ti,ab,kw OR (TKA):ti,ab,kw 10595

#1 MeSH descriptor: [Arthroplasty, Replacement, Knee] explode all trees 3316

**（5）The search strategy used for CNKI:**

(SU%膝关节置换) AND (SU%不对称 OR SU%解剖) AND (SU%平台 OR SU%胫骨 OR SU%假体) 178
