# Supplementary material for: Comparison of the coverage and rotation of asymmetrical and symmetrical tibial components: a systematic review and meta-analysis
Source: BMC Musculoskelet Disord. 2024 Apr 26;25:336. doi: 10.1186/s12891-024-07466-2 (PMC11046884; doi:10.1186/s12891-024-07466-2)
Supplement: Supplementary file 4 — Supplementary Material 4. [file 12891_2024_7466_MOESM4_ESM.docx]

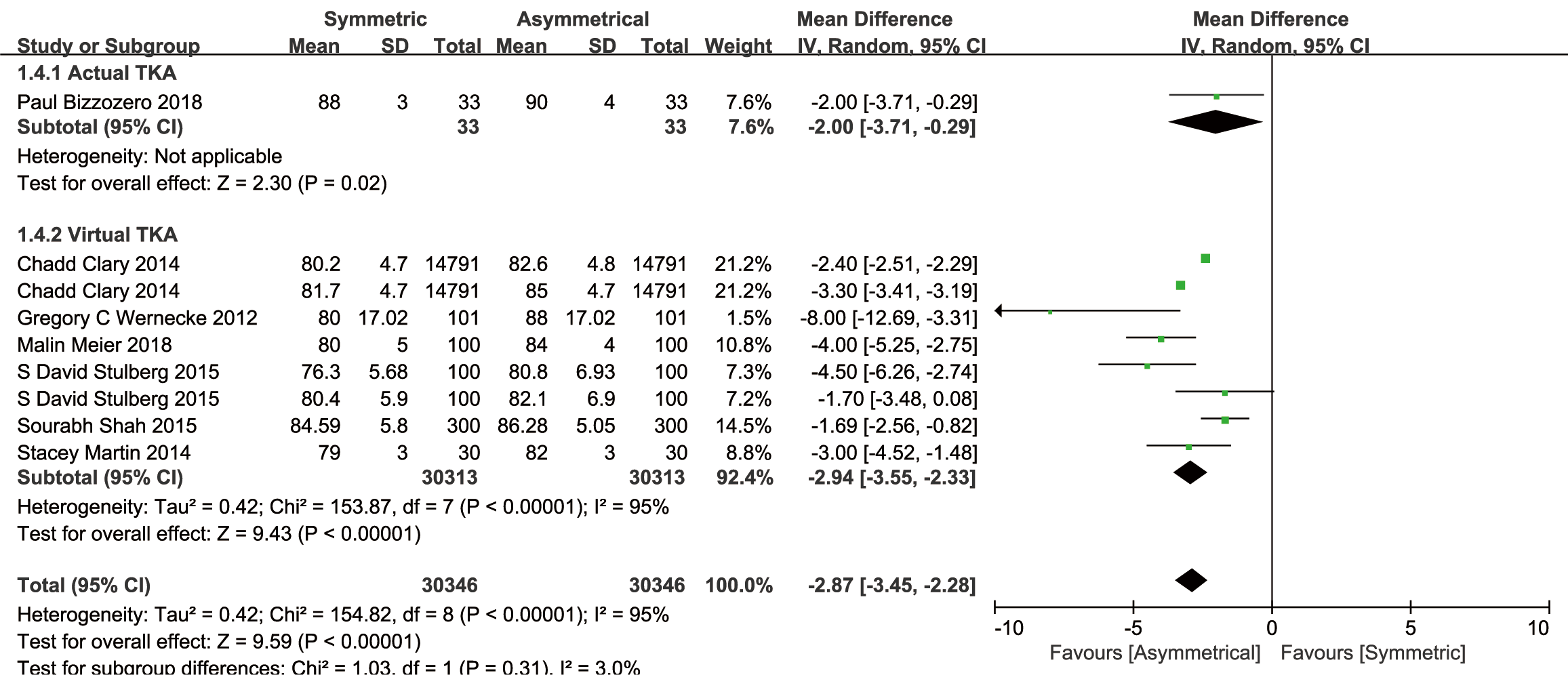


**Supplementary figure S1.**  Subgroup analysis divided into actual TKA or simulated TKA and forest plot for coverage rate
